# Neglected double-J stent with giant bladder stone: a case report

**DOI:** 10.11604/pamj.2021.39.213.29865

**Published:** 2021-07-26

**Authors:** Khoirul Kholis, Muhammad Asykar Palinrungi, Syakri Syahrir, Syarif, Abdul Azis, Stevent Ricardo, Muhammad Faruk

**Affiliations:** 1 Division of Urology, Department of Surgery, Faculty of Medicine, Hasanuddin University, Makassar, South Sulawesi, Indonesia,; 2 Department of Surgery, Faculty of Medicine, Hasanuddin University, Makassar, South Sulawesi, Indonesia

**Keywords:** Double-J stent, ureteral stents, bladder stone, lithotripsy, case report

## Abstract

Double-J (DJ) stents have been widely utilized in urological practice. They are commonly used to relieve ureteral obstruction. Serious complications may occur when stents are left in place for long periods of time. In the present paper, we report a patient with a neglected DJ stent that had been inserted for five years after uterus-tumor surgery and led to a bladder stone. We report a case of a female who presented a bladder stone with a right DJ stent in the pelvic cavity. The stone was evident in radiological examination in an incidental finding. The treatment was transurethral cystolithotripsy. This case reminds us of the necessity of providing enough information and appropriate knowledge pertaining to the insertion of a ureteral stent. Transurethral cystolithotripsy is one of the treatment methods and can be suggested as a definitive method in consideration that it is a clinically effective and safe intervention.

## Introduction

Double-J (DJ) stents have been widely utilized in urological practice [1]. They are commonly used to relieve ureteral obstruction by determining ureteral stent patency due to edema or injury of the ureter [2,3]. However, serious complications may occur when stents are left in place for long periods of time [1]. A bladder stone is the most common manifestation of lower urinary tract disease, and its prevalence is about 5% of all cases of urinary tract diseases [4-7]. In the present paper, we report a patient with a neglected DJ stent that was inserted five years prior for uterus-tumor surgery and led to a bladder stone, in line with the updated consensus-based case report (CARE) guidelines [8].

## Patient and observation

**Patient information:** a 50-year-old woman was checked-up for her poor health condition at the hospital. She complained of a persistent pain when she was urinating over a course of 3 months. She experienced gross hematuria once about 2 months before, but she paid less attention to her health status.

**Clinical findings:** from physical examinations, the general condition of the patient was good, and her vital signs were clinically within the normal limits. In view of her urological status, no abnormalities were found in the right and left costovertebral regions. There was no distention or pain in her bladder.

**Timeline of current episode:** the patient had a history of hysterectomy 5 years prior due to a uterus tumor. Insertion of DJ stents is performed prior to hysterectomy for removal of obstruction and ureter identification. However, she had a lack of information concerning the DJ stent insertion, and she did not regularly control her health status after the surgical intervention.

**Figure 1:**
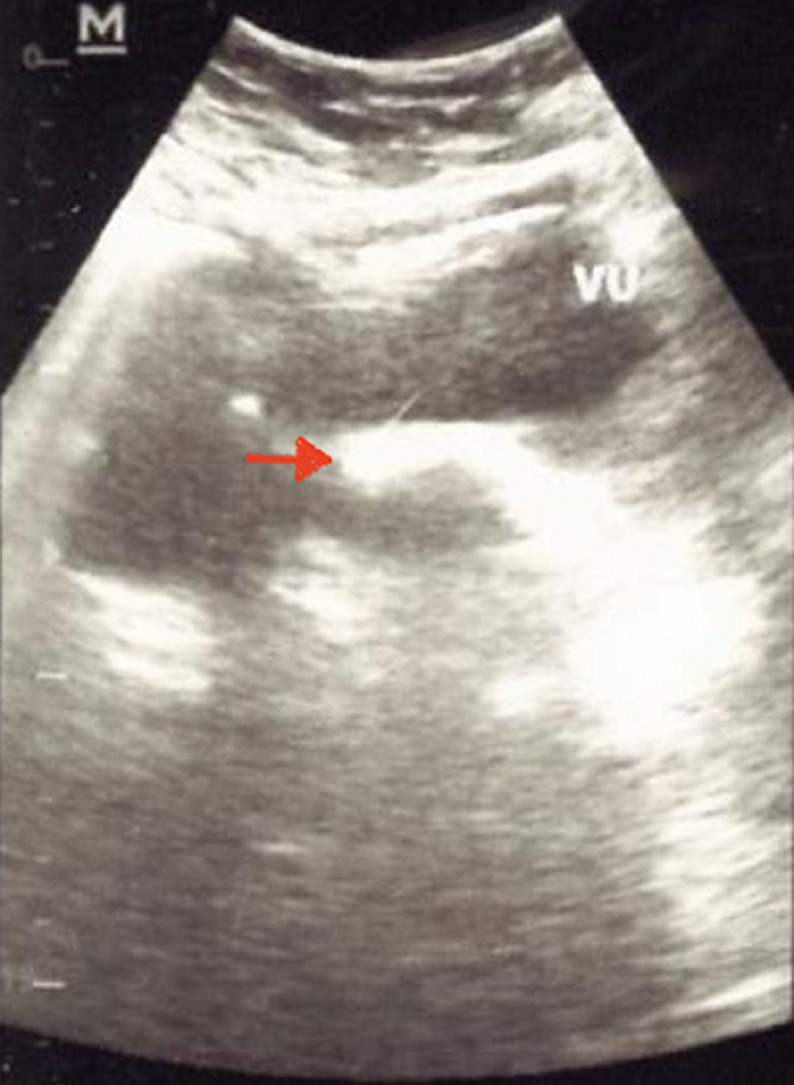
urologic ultrasonography showed a hyperechoic image in the bladder with sizes of 175 mm x 125 mm (red arrow)

**Figure 2:**
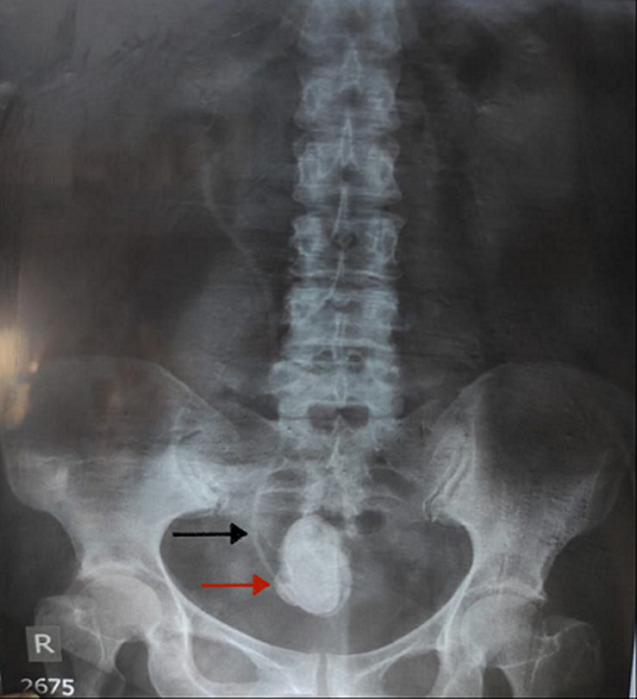
a kidney, ureter, and bladder X-ray showed a radiopaque image in the pelvis with a size of 45 mm x 30 mm (red arrow) and a stent image (black arrow) in the right paravertebral space of the pelvic cavity

**Figure 3:**
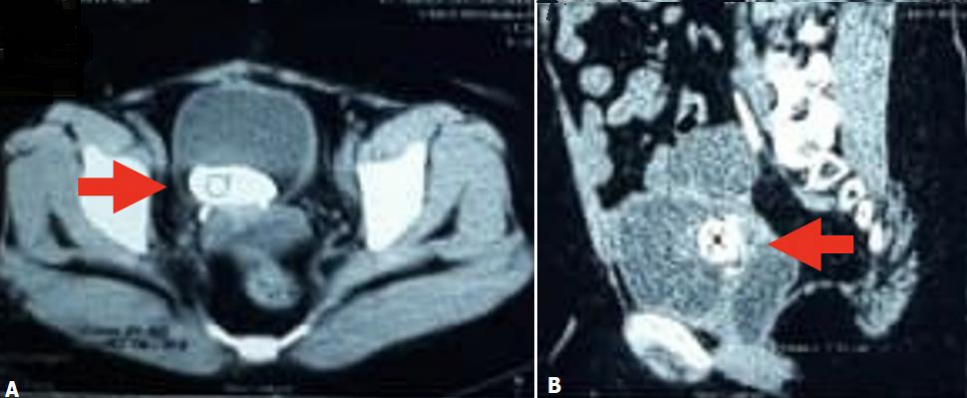
urological computed tomography scan (A, B) showed density of stone in the bladder (arrow) with size of 48 mm x 21 mm x 20 mm and the right ureteral stent in the pelvic cavity

**Diagnostic assessment:** examination with urologic ultrasonography (USG) showed a hyperechoic structure in the bladder with sizes of 175 mm x 125 mm (Figure 1). The size of the left kidney was normal, but dilatation of Pelvi calyceal system (PCS) was detected at the right kidney without stone formation. A photograph of the kidney, ureter, and bladder (KUB) showed a radiopaque image at the pelvic cavity with a size of 45x30 mm and an image of the double-J stent in the right thoracic paravertebral space (Figure 2). A urological computed tomography (CT) scan showed stone density in her bladder with a size of 48x 21x23 mm and the inserted stent in the right ureter (Figure 3). Her blood components were in normal limits.

**Diagnosis:** the patient was diagnosed with neglected double-J stent with giant bladder stone.

**Figure 4:**
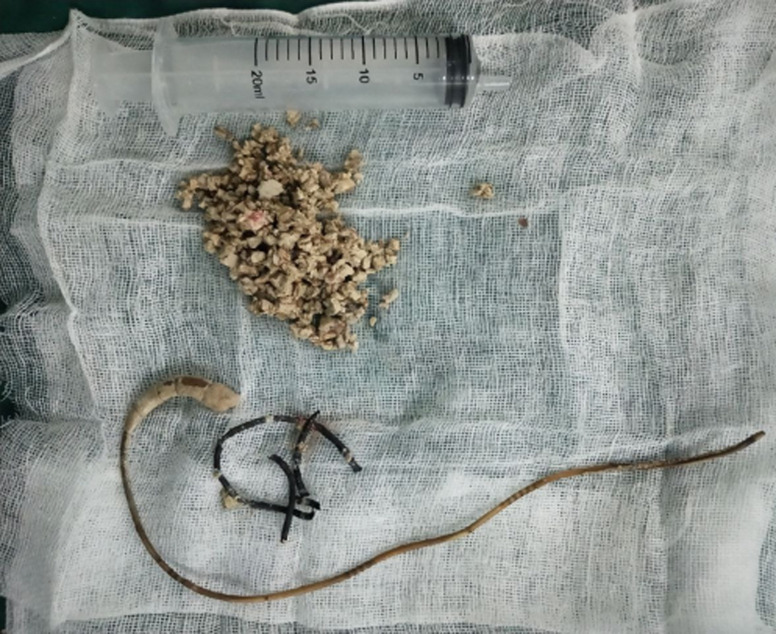
endoscopic removal of the double-J stent and the crushed fragments of bladder stone

**Therapeutic interventions:** transurethral cystolithotripsy was mechanically conducted, and endoscopic removal of the DJ stent was carried out with spinal anesthesia (Figure 4). During the operation, two stones were found with whitish yellow color.

**Follow-up and outcome of interventions:** at the postoperative evaluation 3 month later, the patient´s general condition was satisfactory.

**Patient perspective:** the patient shared their perspective on the treatment in term of micturition stating that they felt no symptoms and could return to their normal activity.

**Informed consent:** the patient provided informed consent for the publication of his clinical data. The presented data are anonymized and risk of identification is minimal.

## Discussion

The use of ureteral stents in surgery was described as early as the 19^th^ century by Shoemaker in 1895 [9]. The first DJ or double pigtail stents were developed almost simultaneously by Finney and Hepperlen [10,11]. Ever since, the use of DJ stents has increased dramatically in urology practice worldwide [12]. In the present case report, insertion of a DJ stent was conducted as a prophylaxis to reduce iatrogenic ureteral trauma before gynecological operation. The DJ stent generally needs to be replaced or removed within 6 weeks to 6 months to avoid complications. Serious complications such as migration, fragmentation, encrustation, and stone formation still occur, especially when stents are left in place for long periods of time [1]. In the present case, the ureteral stent was inserted since 5 years before and led to a bladder stone.

Minor encrustation at the surface of stent sometimes exists, and severe encrustation may occur in a neglected ureteral stent. The indwelling time of a DJ ureteral stent is a risk factor for encrustation [2,13]. According to Shaw *et al*. protein absorptions occur on the stent surface and result in multiple layers of bacterial deposits. They generate an exopolysaccharide matrix in the form of crystal precipitation that leads to encrustation [14]. Neglected stent cases are multifactorial [1]. Patients or their relatives who lack information pertaining to the ureteral stents seem to be an important cause of neglected ureteral stent cases [1,15]. In the present case, the patient gained less information pertaining to the insertion of the ureteral stent when she underwent operation 5 years ago. As a consequence, she did not routinely control the inserted stent. The present study is compatible with other studies of neglected stent cases. A study by Jhanwar *et al*. in India revealed that the factors affecting patients who neglected the control of a ureteral stent were a lack of information (38.16%), poor economic status (23.32%), poor adherence of patients of (19.08%), patients considering it less important to control the inserted stent (12.72%), and lower educational status (6.36%) of the all investigated patients [16].

Clinically detrimental effects in the form of complaints of patients due to neglected DJ stents may vary [1,12]. A retrospective study by Patil *et al*. revealed that the main complaints of patients in descending order were dysuria (80%), irritative lower urinary tract symptoms (53.3%), hematuria (40%), plain pain (30%), and recurrent infection of the urinary tract (26.67%) [1]. A study by Al-Hajjaj *et al*. in Suria also reported intermittent right flank pain and irritative lower urinary tract symptoms [15]. Their studies are consistent with the present case in that dysuria and hematuria were the main complaints of the investigated patient.

## Conclusion

In the present case report, a bladder stone might occur due to neglected DJ stents. A lack of information and appropriate knowledge pertaining to the insertion of the ureteral stent are the main causes of bladder stone cases. Therefore, sharing information before and after the insertion of a ureteral stent and follow-up of the health status of patients are important measures in managing the detrimental clinical effects of inserted ureteral stents in the urinary tract and kidney.

## References

[1] Patil S, Raghuvanshi K, Jain DK, Raval A (2020). Forgotten ureteral double-J stents and related complications: a real-world experience. African J Urol.

[2] Ahallal Y, Khallouk A, El Fassi MJ, Farih MH (2010). Risk factor analysis and management of ureteral double-j stent complications. Rev Urol.

[3] Acosta-Miranda AM, Milner J, Turk TMT (2009). The FECal Double-J: a simplified approach in the management of encrusted and retained ureteral stents. J Endourol.

[4] Francisca Kholis K, Palinrungi MA, Syahrir S, Syarif Faruk M (2020). Bladder stones associated with vesicovaginal fistula: a case report. Int J Surg Case Rep.

[5] Benway BM, Bhayani SB (2012). Lower Urinary Tract Calculi Campbell-Walsh Urol. Medicine.

[6] McQuiston LT, Caldamone AA (2012). Renal infection, abscess, vesicoureteral reflux, urinary lithiasis, and renal vein thrombosis. Pediatr Surg Elsevier.

[7] Palinrungi MA, Syahrir S, Kholis K, Syarif Faruk M (2020). Giant bladder stone formed around sewing-needle in childhood: a case report and literature review. Urol Case Reports.

[8] Riley DS, Barber MS, Kienle GS, Aronson JK, von Schoen-Angerer T, Tugwell P (2017). CARE guidelines for case reports: explanation and elaboration document. J Clin Epidemiol.

[9] Beysens M, Tailly TO (2018). Ureteral stents in urolithiasis. Asian J Urol.

[10] Mosayyebi A, Manes C, Carugo D, Somani BK (2018). Advances in ureteral stent design and materials. Curr Urol Rep.

[11] Aggarwal SP, Priyadarshi S, Tomar V, Yadav SS, Gangkak G, Vyas N (2015). A randomized controlled trial to compare the safety and efficacy of tadalafil and tamsulosin in relieving double-J stent related symptoms. Adv Urol.

[12] Abdelaziz AY, Fouda WB, Mosharafa AA, Abelrasoul MA, Fayyad A, Fawzi K (2018). Forgotten ureteral stents: risk factors, complications and management. African J Urol.

[13] Lin TF, Lin WR, Chen M, Yang TY, Hsu JM, Chiu AW (2019). The risk factors and complications of forgotten double-J stents. J Chinese Med Assoc.

[14] Shaw GL, Choong SK, Fry C (2005). Encrustation of biomaterials in the urinary tract. Urol Res.

[15] Al-Hajjaj M, Kazan MN (2020). Neglected double J stent for 8 Years with giant bladder calculi formation: a case report. Urol Case Reports.

[16] Jhanwar A, Bansal A, Prakash G, Sankhwar S (2017). Endourological management of forgotten double-J ureteral stents: a single centre study. SM Journal Urol.

